# Multicentric Castleman disease with TAFRO syndrome and Sjögren's

**DOI:** 10.1002/ccr3.2502

**Published:** 2019-10-24

**Authors:** Zi Ying Li, Shirley Kim, SiYi Huang, Raza Mian

**Affiliations:** ^1^ Department of Internal Medicine University of California, San Francisco-Fresno Fresno CA USA

**Keywords:** AKI, anasarca, Castleman disease, Sjögren's syndrome, splenomegaly, thrombocytopenia

## Abstract

We describe a patient with Castleman's disease with TAFRO syndrome and concurrent Sjögren's syndrome and investigate whether the autoimmune process may have accelerated the onset of her Castleman's disease. Patient was treated with R‐CVP therapy with remission of symptoms although there is no current standard treatment.

## Graphical abstract

We describe a patient with Castleman's disease with TAFRO syndrome and concurrent Sjögren's syndrome and investigate whether the autoimmune process may have accelerated the onset of her Castleman's disease. Treated with R‐CVP therapy with remission of symptoms although there is no current standard treatment.

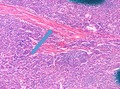

## 1 BACKGROUND

Castleman's disease (CD) is a heterogeneous group of lymphoproliferative disorders and HHV‐8 negative, and idiopathic multicentric CD (iMCD) is one of the rare and life‐threatening subtypes with poorly understood etiology. Vascular endothelial growth factor (VEGF) and interleukin 6(IL‐6) are often found elevated in most of the iMCD cases. A 36‐year‐old Hispanic female with history of seizure disorder presented with symptoms consisted with TAFRO syndrome (thrombocytopenia, anasarca, myelofibrosis, renal failure, organomegaly) with lymph node biopsy confirmed as Castleman's disease. Her ethnicity, age of disease onset, newly diagnosed Sjögren's syndrome, low IL‐6 level at disease presentation and elevated VEGF level despite siltuximab treatment made her hospital course eventful and unique compares to others Castleman's disease cases. We emphasize the importance of further research in reliable prognostic markers and perhaps immunotherapy for patient with HHV‐8 negative/idiopathic MCD and concurrent autoimmune disease.

Castleman's disease (CD) is a group of lymphoproliferative disorders presented as angiofollicular lymph node hyperplasia with polyclonal B lymphocytes expansion from cytokine storm often including IL‐6 and vascular endothelial growth factor (VEGF).^1^ It is divided into unicentric CD (UCD) and multicentric CD (MCD) with histology features of hyaline vascular, plasma cell and mixed, which the hyaline vascular is correlated with UCD, whereas plasma cell is more related to MCD.^2^

Multicentric CD can be further subdivided into human herpes virus‐8 (HHV8) associated MCD, which can be observed in AIDS patients. The etiology for HHV‐8 negative/idiopathic MCD (iMCD) still largely unknown but it could be presented with TAFRO syndrome (thrombocytopenia, anasarca, fever, reticulin fibrosis, organomegaly), which has been mostly observed in Japan.^3^ Interferon‐γ‐induced protein 10 (IP‐10) is more associated with iMCD‐TAFRO syndrome that was found elevated in serum in patient with iMCD‐TAFRO during flare‐ups when compared to serum from healthy individual.^4^

Twenty‐five percent of the new CD cases in the United States are iMCD with median age at diagnosis around 50‐65, and more than 50% are male.^5^, ^6^ A retrospective study in 2014 showed the estimated the 5‐year survival rate in MCD is around 28% less compares to UCD.^7^ The rarity of iMCD also makes it challenge to follow‐up on patient's long‐term response to therapy. A systemic literature review by Sitenga et al^8^ on 7 studies with Caucasian, Asian, and African American iMCD patients demonstrated 5‐year survival rates of 96.4% in siltuximab therapy (anti‐IL6). There is no clinical trial or case study on effective iMCD treatment in Hispanic population yet.

From the study by Anaya et al,^9^ Sjögren's syndrome is a polyclonal lymphoproliferation autoimmune disease with the ability to transform to a monoclonal population with B‐cell hyperstimulation, which could be a precursor of certain malignancy. Sjögren's syndrome has been seen co‐existing with Castleman's disease in other case reports, and iMCD patients could have developed autoimmune manifestation since they do share similar pathogenic mechanism, which had been demonstrated by a retrospective study of CD patient in a Spanish tertiary hospital.^10^ Biologic therapies against B lymphocytes (anti‐CD20) such as rituximab had shown clinical remission in a case of CD with Sjögren's.^11^

The current study presents a case of hyaline vascular variant iMCD with TAFRO syndrome and Sjögren's syndrome with refractory treatment response to anti‐IL6 and anti‐CD20 therapy.

## 2 CASE REPORT

**Figure 1:**
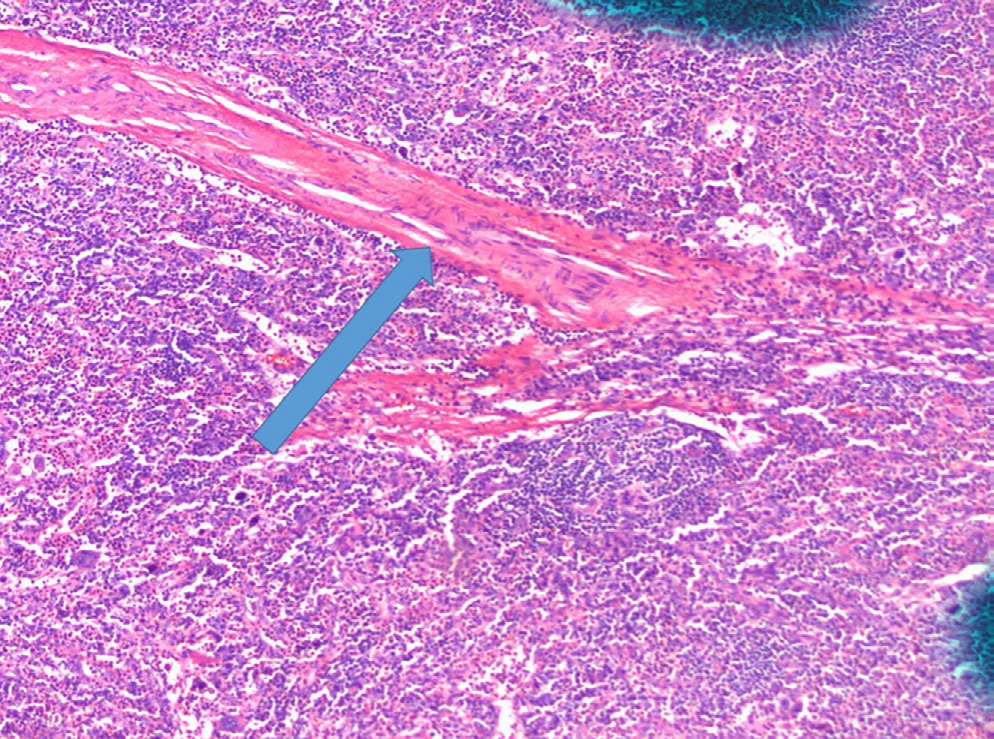
Cervical lymph node in 10× with hyaline vascular variant (arrow)

**Figure 2:**
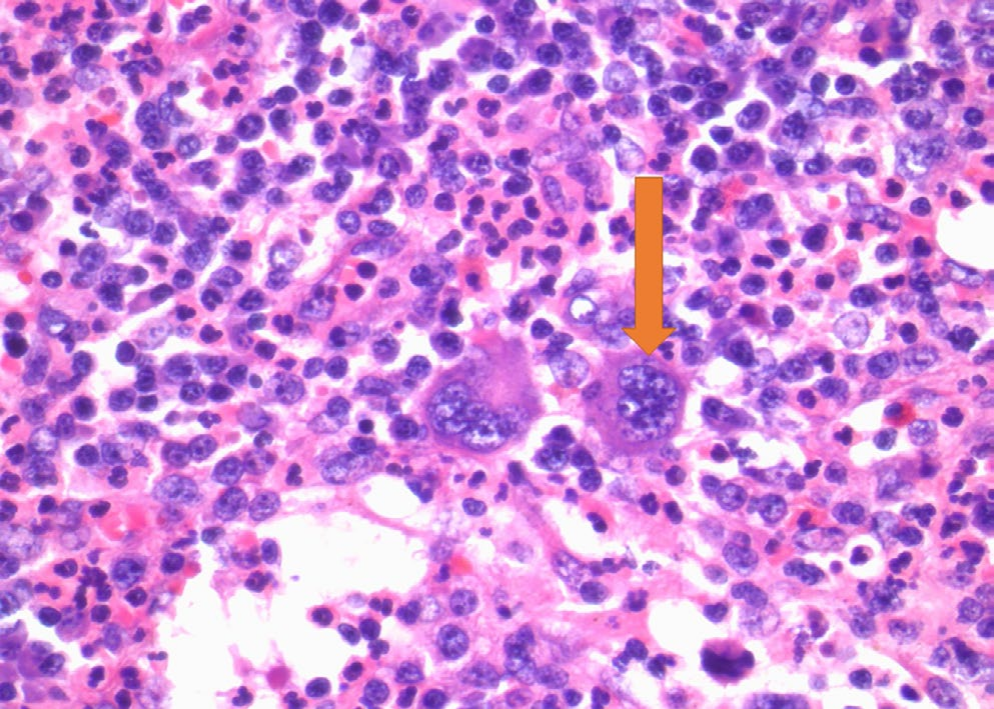
Cervical lymph node in 60× with megakaryocytes and eosinophils (arrow)

A 36‐year‐old Hispanic female with history significant for seizure disorder and hypothyroidism who presented to outside hospital for worsening anasarca, abdominal pain with fever, and dark urine for 3 months. Physical examination was significant for 3 + bilateral pitting at the lower extremities and body trunk. Laboratory findings were consistent with leukocytosis, anemia, thrombocytopenia, kidney insufficiency, proteinuria, and elevated erythrocyte sedimentation rate (ESR). Bone marrow biopsy on October 2018 showed fibrotic change. (Detail See Table 1). Computed tomography (CT) of abdomen and pelvis showed splenomegaly and axillary, bilateral inguinal, and retroperitoneal lymphadenopathy. Cervical lymph node and bone morrow biopsy showed hyaline vascular variant with megakaryocytes. (Figures 1 and 2) She had received IV glucocorticoid and rituximab without improvement.

**Table 1:** Laboratory findings

Test name	Value
Complete Blood Count	White blood count (WBC) −15.48 to 10.55 × 10^3^/µL
	Hemoglobin 10.0 to 7.1 g/dL
	Platelet 74 to 54 × 10^3^/µL
Basic Chemistry Panel	Potassium 5.2 mmol/L
	BUN 43 mg/dL
	Creatinine 1.27 from 0.77 mg/dL baseline 1 mo ago
	eGFR‐ 48
Urine Analysis	Urine protein 500 mg/dL
	Urine blood –2+
	Urine red blood count 10 per high power field (HPF)
	Urine white blood count 8 per HPF
	Urine hyaline cast 66 LPF
	Urine granular cast 32 HPF
Human immunodeficiency virus (IV) antibody and antigen	Negative
Human herpes virus‐8 antibody (HHV‐8)	Negative
Quantiferon‐Tuberculosis (TB)	Negative
Coccidioidomycosis serology	Negative
Chronic hepatitis panel	Negative
Antinuclear antibody (ANA)	1:160 titer, homogenous pattern
SS‐A antibody	IgG >8.0 IgG antibodies
Double strand DNA (dsDNA) antibody	5 IU/mL
24‐h urine immunofixation	Kappa chains 9.94 mg/dL/mg/24 h, lambda chains 4.16 mg/dL/mg/24 h, no monoclonal proteins detected
Bone survey	Negative for bony destructive lesion
Skin biopsy	Negative for glomeruloid hemangioma
Vascular endothelial growth factor (VEGF)	803 pg/mL at admission
Vascular endothelial growth factor (VEGF)	150 pg/mL at discharge
Interleukin 6 (IL‐6)	<5 pg/mL

She was later transferred to current hospital for higher level of care. New laboratory findings included elevated VEGF (803 pg/mL), low IL‐6 (<5.0 pg/mL), elevated C‐reactive protein (CRP), antinuclear antibodies (ANA) 1:160, SS‐A >8.0 IgG antibodies, HHV‐8, HIV and hepatitis panel, 24‐hour urine immunofixation and bone survey were negative.

She was diagnosed with HHV‐8 negative iMCD with TAFRO syndrome. Two cycles of siltuximab (anti‐IL‐6) once weekly and oral prednisone and IV methylprednisolone were given but VEGF level was still persistently elevated at 650 pg/mL. Her hospital course was complicated by seizures, and brain magnetic resonance imaging (MRI) was consistent with posterior reversible encephalopathy syndrome (PRES). Rheumatology was consulted due to the elevated ANA titer and SS‐A antibodies and diagnosed Sjögren's syndrome. No additional Sjögren's syndrome treatment was given since patient was already on glucocorticoid without any complaints of dry mouth or eye.

Due to the concern for hypertension and PRES, R‐CVP therapy (Rituximab, Cyclophosphamide, Vincristine, and Prednisone) was initiated later and VEGF down‐trended to 150 pg/mL. She was sent home with follow‐up with Hematology without oral prednisone taper.

## 3 DISCUSSION

In the above case, our patient had clinical presentation for HHV‐8 negative iMCD with TAFRO syndrome (thrombocytopenia, anasarca, fever, bone marrow fibrosis, splenomegaly, and renal insufficiency). She did have an atypical iMCD presentation based on her age group, gender, and hyaline vascular variant in lymph node biopsy, which is usually seen in UCD. Females between the ages of 25‐44 years old were rare in a CD study conducted by Robinson et.al.^7^ This leads to whether a concurrent autoimmune disease could have accelerated the onset of CD by increasing the rate of lymph node hyperplasia due to chronic inflammatory status and similar pathogenic mechanism. HHV‐8 negative iMCD with TAFRO syndrome with or without Sjögren's syndrome cases were reported in Japan and other countries, and it was rarely reported in literature on Hispanic population.^12^ This not only makes our case unique among other CD cases, but it also unveils interesting future research ideas in iMCD, especially with the possibility in different ethnicity groups in terms of disease diagnosis and treatment response.

The IL‐6 level in this case was not as elevated as it was seen in other iMCD cases and it could be an indication for iMCD flare‐ups. A study consisted of 11 blood samples from iMCD patients during flare‐up showed low IL‐6 level, and pro‐inflammatory cytokine like interferon‐γ‐induced protein 10 (IP‐10) and tumor necrosis factor (TNF)‐α were found elevated.^13^, ^14^

Since our patient has HHV‐8 negative iMCD with TAFRO syndrome, IP‐10, and TNF‐α could have been the potent prognostic and diagnostic markers for her therapy response instead of relying on VEGF level alone. These could correlate on how our patient's VEGF level failed to drop after 2 cycles of siltuximab therapy; perhaps we did not use the appropriate disease activity marker for therapeutic response to begin with, but further researches need to be done to confirm this speculation.

Another cohort study by Liu et al^15^ was able to express the presence of inhibitor of differentiation 1 (ID1) marker in human tumor cell, which is another activator in the signaling pathway to increase VEGF production. This may open other modalities for as there are multiple cytokines to target and block lymph hyperplasia in the case of iMCD. Interleukin‐1 (IL‐1) is also found to have the ability to promote IL‐6 production, and anti‐IL1 had shown therapeutic effect in an iMCD patient without autoimmune disease but refractory to steroid, anti‐IL6, anti‐CD20, and anti‐TNF‐α therapies.^16^

The above cytokines that we mentioned, IL‐1, IP‐10, TNF‐α, and ID‐1, could be the next diagnostic and prognostic markers and potent biologic therapy candidate for iMCD in patient with co‐existing autoimmune disease but further research with larger patient population will be urgently required.

Diagnosing iMCD could be challenged to most of the physician especially when patient's clinical presentation overlaps with other autoimmune diseases. There are published diagnostic criteria for HHV‐8 negative iMCD, which was based on review of 244 clinical cases and 88 tissue samples.^17^ After diagnosing, patients could be properly treated or referred to appropriate subspecialty for further evaluation or treatment. Patients are encouraged to enroll ACCELERATE natural history research study for CD patient by the Castleman Disease Collaborative Network, which could further empower future iMCD researches.^18^

## 4 CONCLUSION

We reported a rare case of hyaline vascular variant HHV‐8‐negative idiopathic multicentric Castleman disease with TAFRO syndrome and Sjögren's syndrome. The usual elevated IL‐6 level and prompted clinical response to anti‐IL‐6 therapy were not observed in our case. Further studies in varied ethnicity groups are mandated to identify the other potent disease activity and prognostic markers and target therapy in iMCD with co‐existing autoimmune disease.

## CONFLICT OF INTEREST

No conflict of interest.

## AUTHOR CONTRIBUTIONS

ZYL: contributed to the draft and revision of the manuscript. SK: contributed to the review of the manuscript. SYH: contributed to the review of the manuscript. RM: contributed to the review of the manuscript.

## ETHICAL APPROVAL

This is a single case report and ethnics approval is not required.

## CONSENT

Informed consent was obtained from the patient during admission. A copy of signed consent is submitted.
